# Type I Interferon Impairs Specific Antibody Responses Early during Establishment of LCMV Infection

**DOI:** 10.3389/fimmu.2016.00564

**Published:** 2016-12-05

**Authors:** Matthieu Daugan, Armstrong Murira, Barbara C. Mindt, Amélie Germain, Esther Tarrab, Pascal Lapierre, Jörg H. Fritz, Alain Lamarre

**Affiliations:** ^1^Immunovirology Laboratory, Institut national de la recherche scientifique (INRS), INRS-Institut Armand-Frappier, Laval, QC, Canada; ^2^Complex Traits Group, Department of Microbiology and Immunology, McGill University, Montréal, QC, Canada; ^3^Complex Traits Group, Department of Physiology, McGill University, Montréal, QC, Canada

**Keywords:** LCMV, interferon type I, antibody formation, immunopathology, neutralizing antibodies

## Abstract

Elicitation of type I interferon (IFN-I) has been shown to both enhance and impair cell-mediated immune responses in acute and persistent viral infections, respectively. Here, we show that, in addition to its effect on T cells, IFN-I drives impairment of specific antibody responses through interaction with B cells in the acute phase of lymphocytic choriomeningitis virus (LCMV) infection. This impairment was limited to the T cell-dependent B cell response and was associated with disruption of B cell follicles, development of hypergammaglobulinemia (HGG), and expansion of the T follicular helper cell population. Antigen-specific antibody responses were restored by ablation of IFN-I signaling through antibody-mediated IFN-I receptor blockade and B cell-specific IFN-I receptor knockout. Importantly, IFN-I receptor deficiency in B cells also accelerated the development of LCMV neutralizing antibodies and alleviated HGG. These results provide a potential therapeutic target toward efficient treatment measures that limit immunopathology in persistent viral infections.

## Introduction

The humoral immune response plays a central effector role against viral infection whereby induction of effective antibody (Ab) responses serves as an important correlate toward pathogen clearance. However, during persistent viral infections, e.g., with human immunodeficiency virus (HIV), hepatitis C virus (HCV), or the murine infection model lymphocytic choriomeningitis virus (LCMV), emergence of neutralizing Abs (nAbs) against these highly mutable viruses is delayed, initially narrow in specificity and ineffective against the established infection; as such, the Ab response bears negligible impact on the progression of the disease (1). Accompanying the delayed induction of nAbs, infected hosts also exhibit an altered immunological milieu that features aberrancies to the humoral response such as: (i) dysregulation of B cell subpopulations (2, 3); (ii) hypergammaglobulinemia (HGG) (4, 5); (iii) increase of polyreactive Abs (6, 7); and (iv) impaired response to vaccines (4, 8). Altogether, these perturbations result in a diminished antigen-specific Ab response and an enhanced non-specific polyclonal response. Notably, these immunomodulatory effects are driven directly by viral pathogenic mechanisms and indirectly through immunopathogenesis triggered by host antiviral responses (9). Presently, it is yet to be determined whether this immunological disruption occurs as a function of chronicity or due to mechanisms initiated during the acute stage of the viral infection.

Concomitant with dysregulation of the humoral immune response, an increase in T follicular helper (T_FH_) cells has also been observed during persistent HIV (10) and HCV (11) infections as well as the chronic phase of LCMV infection (12). In the LCMV model, expansion of T_FH_ cells has been attributed to polarization of the CD4 T cell compartment toward T_FH_ responses, which suggests a role of cytokines such as type I interferon (IFN-I) that skew differentiation and maturation toward T_FH_ and away from T helper type 1 (T_H_1) cells (13). The role of IFN-I signaling with respect to T cells is well characterized and increasing evidence shows that this antiviral cytokine has both enhancing and immunosuppressive effects on the T cell response upon viral infection (14, 15). Two recent studies clearly outlined the bipolar effect that IFN-I renders on T cell-mediated immune responses by comparing the expression profile of IFN-I and IFN-stimulated genes (ISGs) in LCMV Armstrong (acute) versus LCMV Clone 13 (Cl13; persistent) infection (16, 17). Collectively, this research revealed that although protective upon transient elicitation such as in acute infections, prolonged elevation of IFN-I levels postinfection led to immunosuppression of T cell responses. In these studies, sustained expression of IFN-I was shown to drive upregulation of immunosuppressive molecules such as PD-1 and IL10 as well as disruption of splenic architecture and dampened effector CD8+ T cell (CTL) responses (16–21). Altogether, this contributes to the failure of viral clearance and eventual persistent infection.

Similarly, IFN-I production has also been shown to enhance the development of the Ab response against acute viral infections or vaccine antigens (22–27). Akin to T cells, the effect of IFN-I on B cell responses has been shown to drive increased cellular activation and class switching recombination (CSR) in the T-cell-dependent arm of the humoral immune response (23, 25, 27–29). The upregulation of ISGs in B cells from HIV-viremic patients (30) is also indicative of a role played by IFN-I during chronic infections.

However, unlike the deleterious role played by the cytokine against T-cell responses during persistent infection, the effect of IFN-I on B cell responses in this context is yet to be fully elucidated. In this report, we use the LCMV mouse model to further characterize the molecular mechanisms that drive the modulation and resulting humoral immune dysregulation during persistent virus infection.

## Results

### LCMV Infection Impairs the Humoral Response to T-Dependent Antigens

**Figure 1:**
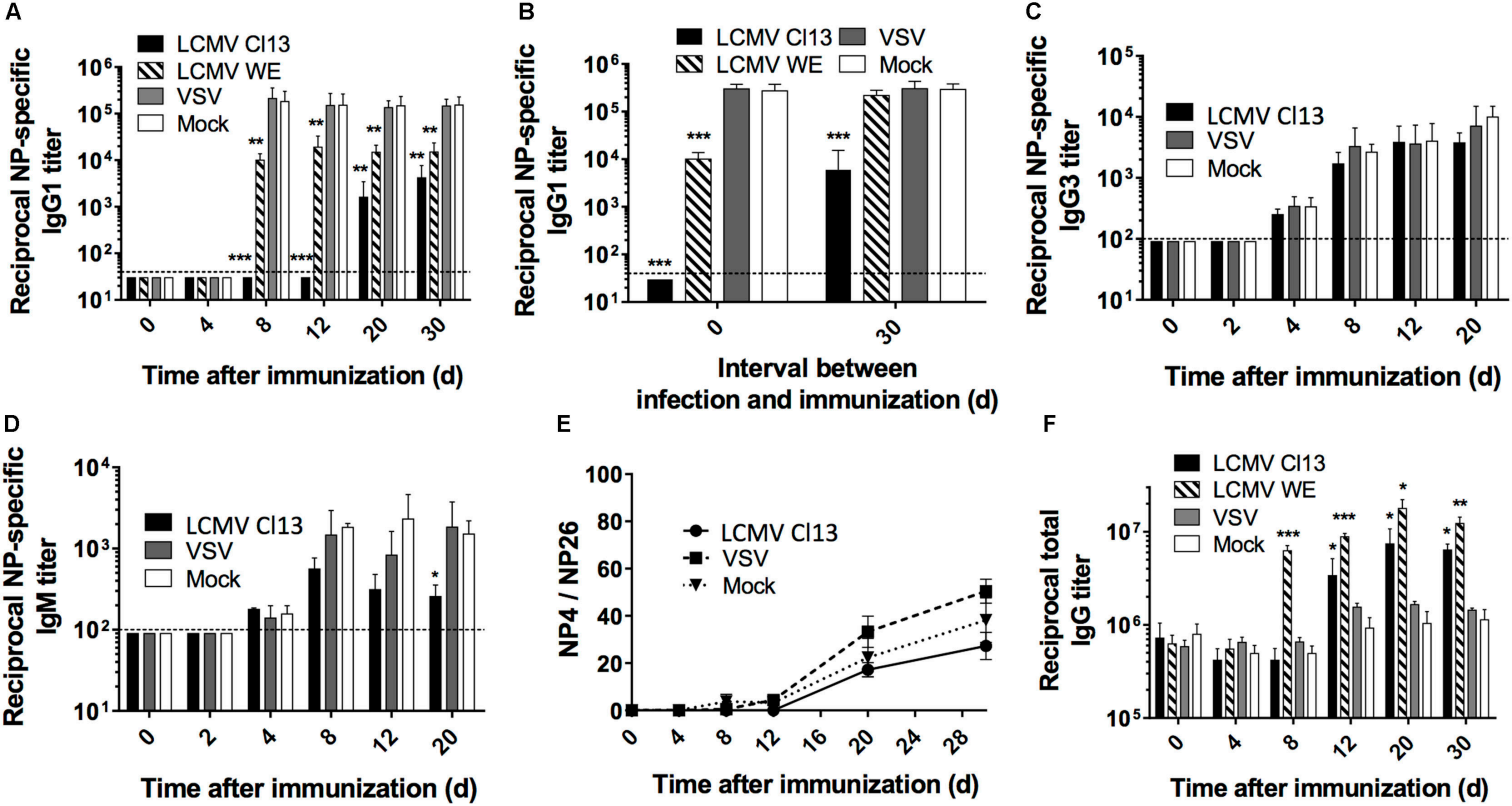
**LCMV infection impairs the NP-specific Ab response in a T cell-dependent manner**. B6 mice (four per group) were infected with LCMV Cl13 (black), LCMV WE (hatched), VSV (gray), or mock infected (white). Mice were immunized the same day (except when indicated otherwise) with an i.p. injection of NP_53_-CGG in alum **(A,B,E,F)** or NP_40_-FICOLL in PBS **(C,D)**. **(A)** NP-specific IgG1 response monitored by ELISA following NP_53_-CGG immunization. **(B)** Mice were infected as above and immunized with NP_53_-CGG the same day (d0) or 30 days after infection (d30), and IgG1 NP-specific responses were monitored by ELISA on d8 postimmunization. NP-specific IgG3 **(C)** or IgM **(D)** responses monitored by ELISA following NP_40_-FICOLL immunization. **(E)** ELISA plates were coated with NP_4_-BSA or NP_26_-BSA and high affinity Ab responses were measured as a ratio of Abs binding to NP_4_-BSA versus the total anti-NP IgG1 response binding to NP_26_-BSA. **(F)** Total serum IgG responses following concomitant infection and NP_53_-CGG immunization. Statistical analysis was performed by individual *T*-tests between experimental groups and the mock-infected group. *p < 0.05, **p < 0.01, ***p < 0.001. The dotted line represents detection threshold. **(A–E)** Representative of two independent experiments. **(F)** Representative of four independent experiments.

Although the influence of escape mutations within the glycoprotein envelope of LCMV as well as dysregulated T cell responses have been implicated in the late appearance of nAbs (31–33), it is unclear whether broader modulation of the immune response also contributes to the disrupted Ab response. To directly evaluate this, we analyzed the Ab response against the model T-dependent (TD) antigen, nitrophenyl (NP) coupled to chicken gamma globulin (CGG) in the context of CTL-controlled LCMV (34, 35), and Ab-controlled vesicular stomatitis virus (VSV) infection (36). The focal point of these experiments was based on the NP response rather than comparing the antiviral response to clearly distinguish and determine modulation to the global immune response independent of LCMV versus VSV whose pathogenic determinants drive distinctive responses. C57BL/6 (B6) mice were infected with either LCMV Cl13 or LCMV WE (acute); VSV Indiana or mock infected with culture media only. All groups were contemporaneously immunized with NP-CGG, which predominantly elicits an IgG1 response (37). At various time points after immunization, the NP-specific IgG1 serum response was monitored by ELISA, which revealed that Ab titers were drastically reduced in LCMV Cl13-infected mice, compared to VSV-infected or mock-infected control mice (Figure 1A). Furthermore, this impairment was also present albeit to a lesser extent in mice infected with LCMV WE (Figures 1A,B, left panel). Although LCMV-Cl13-associated impairment of NP-specific responses declined after day (d)12, the increase in the IgG1 responses thereafter did not attain the levels observed in VSV-infected or mock-infected groups for the duration of the experiment (30 days) (Figure 1A). The kinetics of the disrupted NP-specific response and the impact by both the acute and persistent strains of LCMV suggest that the immunological process that drives this phenotype occurs early after infection whereas the Cl13 strain featured more adverse impairment due to viral persistence. Upon infection with LCMV Cl13 or VSV as above and simultaneous immunization with a T-independent Type 2 (TI-2) antigen, NP-FICOLL, NP-specific IgG3 (Figure 1C), and IgM (Figure 1D) responses were similar in all groups although a trend toward weaker IgM responses in the LCMV-infected group was observed at latter time points. These results demonstrate that LCMV predominantly impairs the TD response. Similar results were also observed in LCMV WE-infected mice (data not shown). Despite the impairment of the TD response, however, the affinity maturation process was unaltered by LCMV Cl13 infection. As illustrated in Figure 1E, the ratio of high affinity anti-NP IgG1 Abs binding to NP_4_-BSA versus the total anti-NP IgG1 response, measured using NP_26_-BSA, reflected a similar increase in Ab affinity at various time points in all three experimental groups. Again, similar results were observed for LCMV WE (data not shown). Thus, although diminished in serum concentration, the quality of the NP-specific response was not affected by LCMV infection. Importantly, the reduction in the NP-specific IgG1 response occurs in the context of increased total IgG serum levels that is evident by d12 in LCMV Cl13- and d8 in LCMV WE-infected mice compared to VSV- or mock-infected animals (Figure 1F). These results are in agreement with the emergence of polyclonal non-specific B cell activation and resultant HGG that is observed during LCMV infection (38) and other persistent viral infections such as HIV (4, 6) or HCV (39).

To further characterize the duration of LCMV-associated effects on the NP-specific response, we immunized mice 4 days before (d-4) and on d4, d8, d12, d20, or d30 after LCMV WE or VSV infections (Figure S1 in Supplementary Material). In this particular experiment, the kinetics of viral clearance within a limited window are important to facilitate accurate assessment of the effect of Ab-response impairment by LCMV. As such, the acute strain, LCMV WE, was used given that this variant is cleared from lymphoid organs within about a week (35). As shown in Figure S1A in Supplementary Material, the Ab response in the VSV-infected group was unaffected irrespective of the time interval between infection and immunization. On the other hand, NP-specific IgG1 responses in LCMV WE-infected mice revealed similar levels of impairment upon immunization on d4, d8, and d12 postimmunization. However, the impact on the NP-specific response upon immunization on d4 before or d20 after infection was less severe relative to the other time points. This indicates that B cell responses: (i) were less susceptible to LCMV impairment if established prior to infection; (ii) start to recover by d20 following LCMV WE infection before returning to normal levels by d30 postinfection; and (iii) remain impaired past d30 following infection with LCMV Cl13 (Figure 1B, right panel). Finally, changes in affinity maturation did not attain statistical significance irrespective of the time interval between LCMV infection and immunization (Figure S1B in Supplementary Material). Taken together, these results indicate that LCMV impairs the development of specific Ab responses early following establishment of infection, which is sustained in the context of persistent infection.

### LCMV Infection Modifies the Lymphoid Microenvironment and B Cell Function

**Figure 2:**
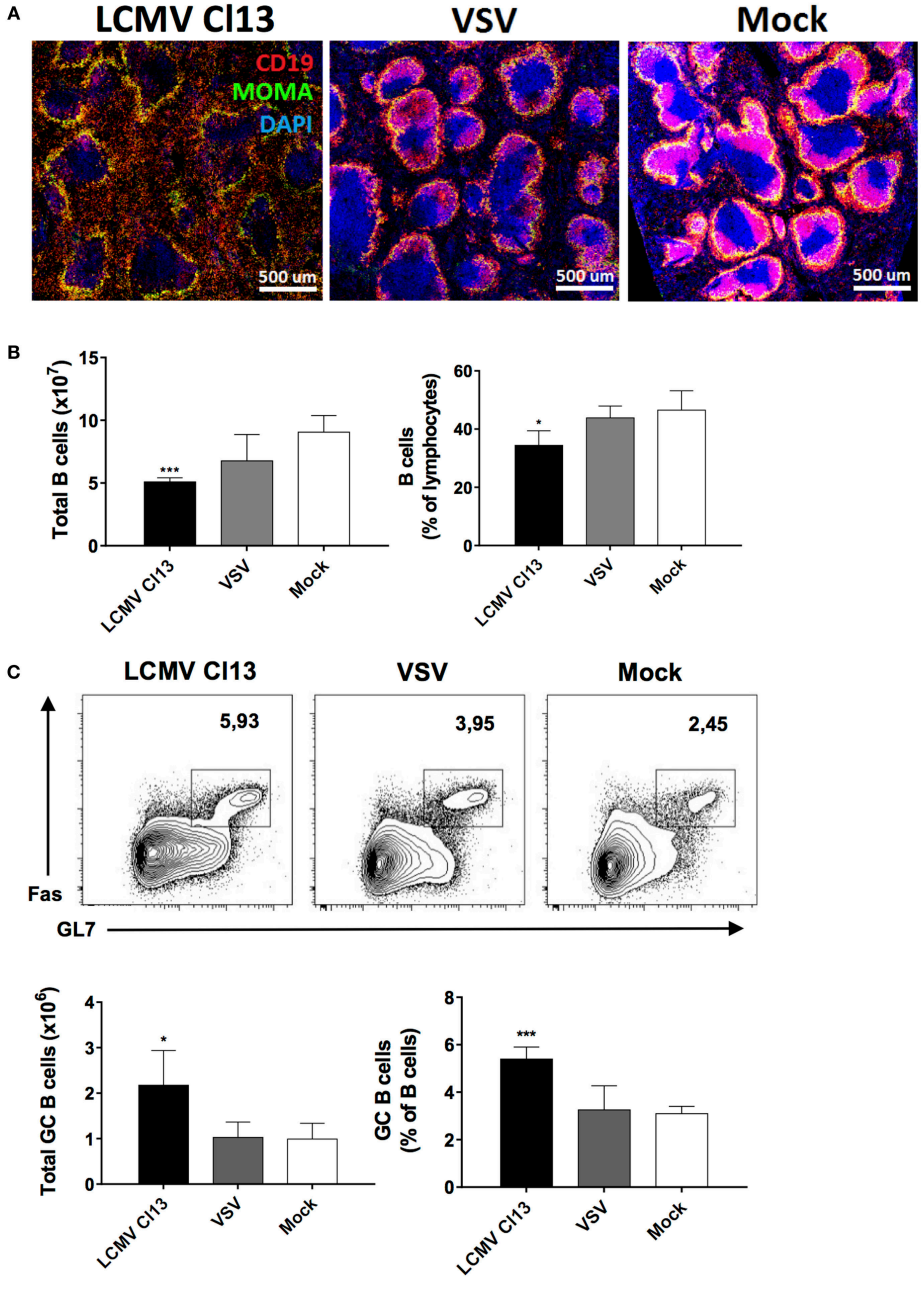

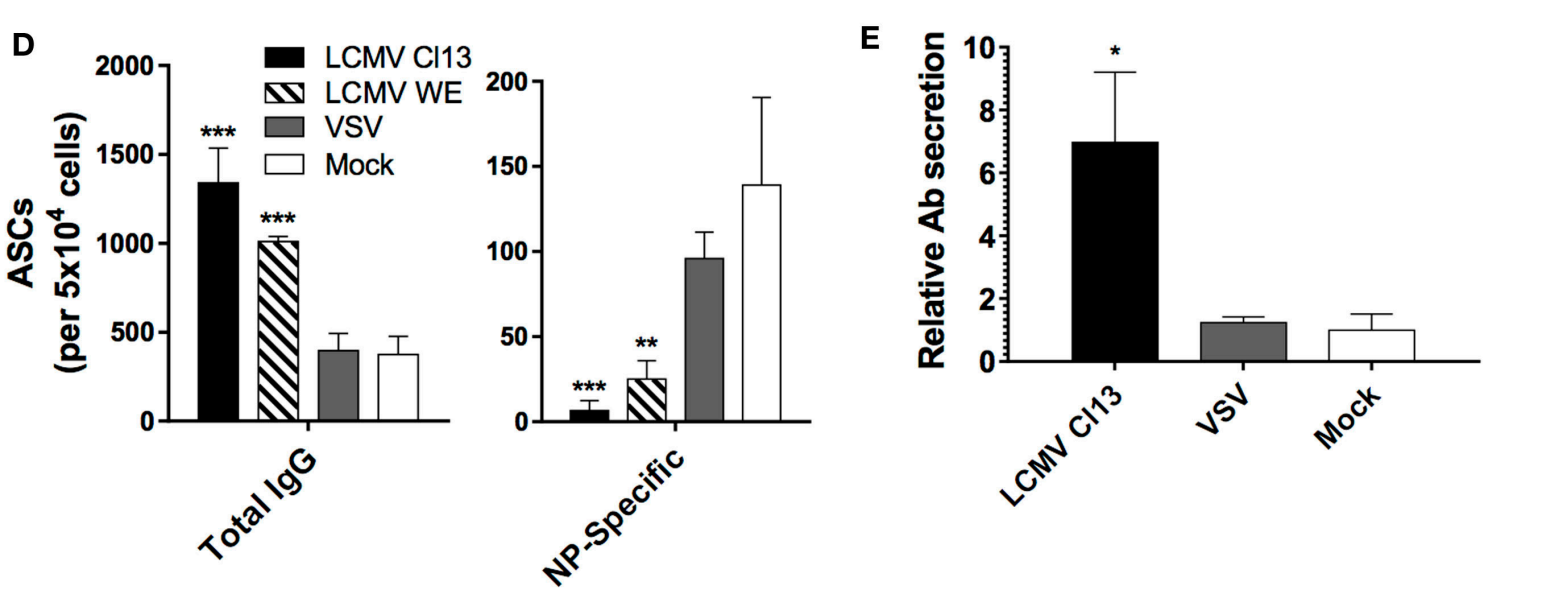
**LCMV infection disrupts the splenic follicular architecture while increasing GC B cell numbers and non-specific Ab responses**. B6 mice (four per group) were infected with LCMV Cl13 (black), LCMV WE (hatched), VSV (gray), or mock infected (white). Mice were immunized the same day with an i.p. injection of NP_53_-CGG in alum and TD B cell responses were analyzed on d8 after infection. **(A)** Immunofluorescence showing CD19 (red), MOMA-1 (green), and DAPI (blue) expression on spleen sections. **(B)** Total B cell numbers and proportions were enumerated by flow cytometry. **(C)** Number and proportion of splenic GC B cells. **(D)** Number of total and NP-specific IgG-secreting cells detected by ELISPOT. **(E)** Relative Ab secretion of ASCs calculated by *ex vivo* measurement of secreted Abs produced by 10^5^ splenocytes. Statistical analysis was performed by individual *T*-tests between experimental groups and the mock-infected group. *p < 0.05, **p < 0.01, ***p < 0.001. **(A)** Representative of two independent experiments. **(B–E)** Representative of four independent experiments.

To gain further insight into the immunological milieu within which the impairment of TD NP-specific Ab responses occurred, we evaluated B cell populations and the splenic microenvironment in the three infection groups on d8 postinfection/immunization. Similar to a previous report (40), we observed perturbations in the splenic architecture whereby immunohistochemistry revealed a dramatic disruption of B cell follicles in LCMV-infected mice relative to VSV- and mock-infected controls with B cells being mostly found outside of the follicles (Figure 2A). Counterintuitive to this disruption, while total numbers and proportions of splenic B cells were contracted in the LCMV Cl13-infected group (Figure 2B), the number and proportion of GC B cells were significantly increased (Figure 2C). Expansion of the GC B cell population suggested enhanced activation of B cells in the LCMV group. Given that this phenomenon could lead to increased differentiation into effector B cell subsets [e.g., plasma cells (PCs)], we evaluated the splenic Ab-secreting cell (ASC) population on d8 after infection and immunization with NP-CGG using ELISPOT (Figure 2D). In agreement with the expansion of GC B cells and the presence of HGG, the total number of IgG-secreting cells in the LCMV Cl13-infected group was elevated. Conversely, the quantity of NP-specific IgG-secreting cells was significantly decreased in the LCMV Cl13 group, correlating with the depressed antigen-specific Ab response. Similar results were also observed in the bone marrow compartment to which the PCs migrate after differentiation in the secondary lymphoid organs (data not shown) and these features were observed in LCMV WE-infected animals as well (Figure 2D). Last, consistent with the increase in the number of IgG-secreting ASCs due to polyclonal B cell activation during LCMV infection (38), ASCs from this group also displayed an increased secretory capacity (Figure 2E). Expressed here as a ratio, the *ex vivo* Ab concentration from splenic ASCs was quantified from culture supernatants, which revealed that following LCMV Cl13 infection, ASCs secreted a significantly higher quantity of Ab relative to VSV- or mock-infected mice.

### LCMV Infection Triggers the Expansion of T_FH_ and an Increase in Their Effector Function

**Figure 3:**
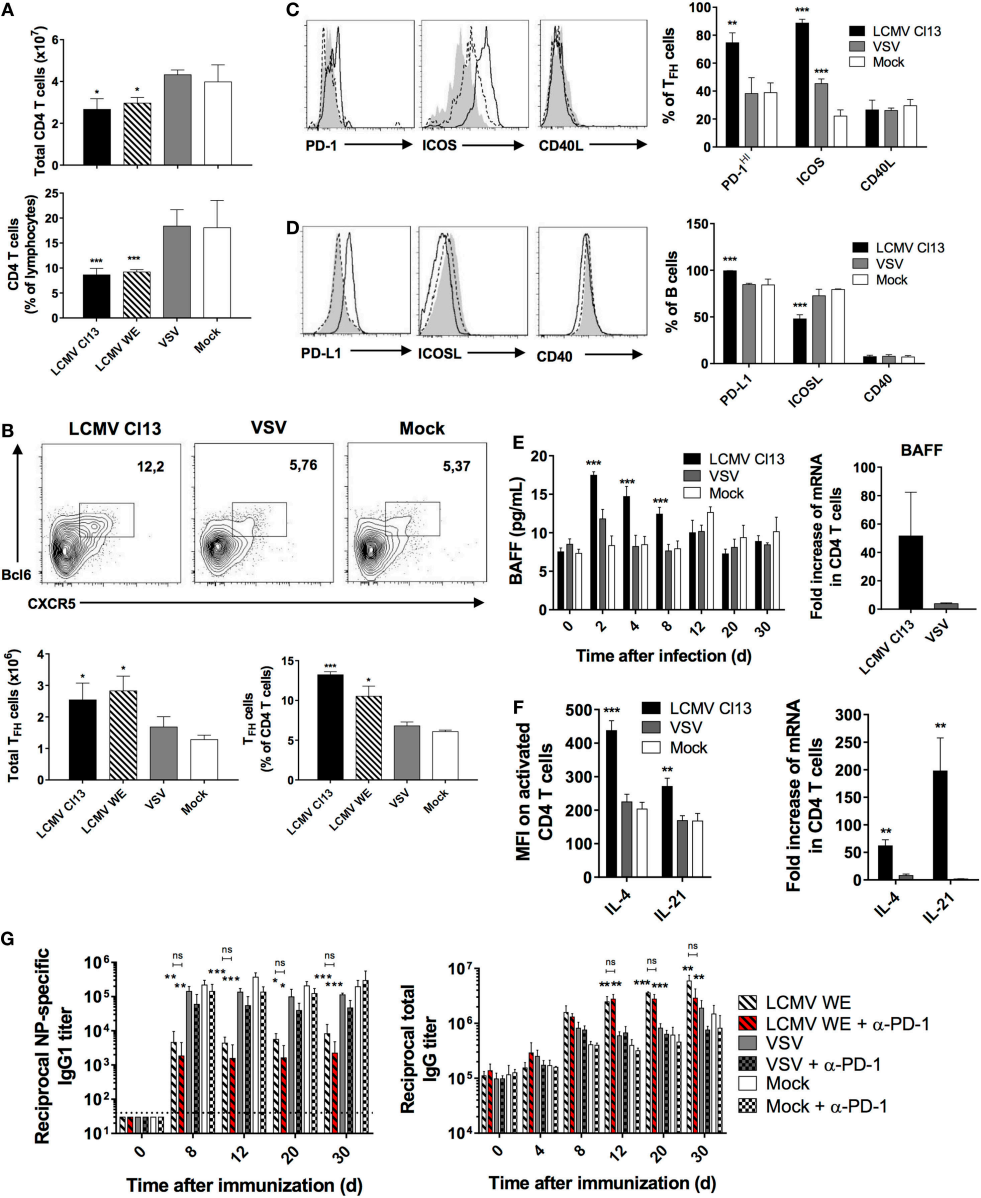
**LCMV infection triggers the expansion of T_FH_ cells and induces their expression of B cell-activating cytokines**. B6 mice (four per group) were infected with LCMV Cl13 (black bars or lines), LCMV WE (hatched), VSV (gray bars or dotted lines), or mock infected (white bars or shaded area). Mice were immunized the same day with an i.p. injection of NP_53_-CGG in alum and CD4^+^ T cells were analyzed on d8 after infection. **(A)** Total CD4^+^ T cell numbers (top panel) and proportions (bottom panel) determined by flow cytometry. **(B)** T_FH_ cell numbers and proportions determined by flow cytometry. **(C)** Proportion of T_FH_ cells expressing PD-1, ICOS, and CD40L and **(D)** B cells expressing PD-L1, ICOSL, and CD40 determined by flow cytometry. **(E)** Serum BAFF concentration (left panel) and BAFF mRNA expression in total splenic CD4 T cells (right panel) measured using ELISA and qRT-PCR, respectively. **(F)** Intracellular cytokine levels (left panel) and mRNA expression (right panel) of IL-4 and IL-21 in CD4 T cells measured using flow cytometry and qRT-PCR, respectively. **(G)** NP-specific IgG1 response (left panel) and total IgG response (right panel) monitored upon infection with LCMV WE (hatched bars), VSV (gray), or mock-infected (white) infection along with injection of PD1-blocking Ab (red hatched or checkered bars). Statistical analysis was performed by individual *T*-tests between experimental groups and the mock-infected group and between non-treated and anti-PD1 Ab-treated LCMV-infected groups when indicated with brackets. *p < 0.05, **p < 0.01, and ***p < 0.001. **(A,B)** Representative of four independent experiments. **(C–E,G)** Representative of two independent experiments. **(F)** Representative of three independent experiments.

Upon phenotypic characterization of the CD4 T cell compartment, we observed that there was a contraction in the absolute number of CD4 T cells (Figure 3A, top panel), which was more evident in the proportion of CD4 T cells relative to total lymphocytes in the LCMV-infected groups (Figure 3A, bottom panel). This coincided with a significant increase in the T_FH_ compartment (CD4^+^CD62L^−^CD44^+^CXCR5^+^Bcl-6^+^) as shown in Figure 3B. These results are supported by previous work, which demonstrated that LCMV increased differentiation of CD4 T cells into T_FH_, and this redirected differentiation program was sustained in LCMV Cl13 due to viral persistence (12). Based on these changes in the T_FH_ population and their potential influence on the humoral immune response, we sought to determine whether the essential costimulatory and signaling molecules that comprise interaction between T_FH_ and GC B cells were similarly modified during LCMV infection. Using phenotypic analysis by flow cytometry, we analyzed the expression of PD-1, which has been shown to be an ideal marker to distinguish GC from non-GC T_FH_ (41). Here, we observed a significantly higher proportion of T_FH_ cells expressing increased levels of PD-1 in LCMV Cl13-infected mice (Figure 3C) indicating a higher number of GC T_FH_ relative to the other cohorts. Likewise, proportions of T_FH_ expressing high levels of ICOS were also significantly elevated relative to mock-infected controls, which was also the case for VSV-infected mice albeit to a lesser extent. Surprisingly, while proportions of B cells expressing PD-L1 similarly increased (Figure 3D), those expressing ICOSL were reduced whereas the ligand pair CD40:CD40L remained unchanged across the three infection groups (Figures 3C,D). Serum levels of BAFF were also elevated in LCMV Cl13-infected mice along with BAFF, IL-21, and IL-4 mRNA and protein expression in CD4 T cells (Figures 3E,F). As previously mentioned, predilection toward T_FH_ differentiation in the context of persistent infection can occur as a result of prolonged expression of IFN-I. However, whether the effects on the humoral response are solely due to a modulated T_FH_ response shaping the B cell response or more direct impact of IFN-I on the B cells is unknown. To determine the potential role of T_FH_ immunomodulation on perturbation of the humoral response, we administered blocking Abs against PD-1, which comprises a key molecular interaction between GC B cells and T_FH_ (42). The LCMV WE strain was used here given that interfering with the PD-1 pathway during the early phase of systemic LCMV Cl13 infection has been shown to induce lethal CD8 T cell-mediated immunopathology (18, 43). As illustrated in Figure 3G, blockade of PD-1 prior to infection and immunization with NP did not alter the NP-specific and total IgG titers relative to the untreated control group. Similar results were obtained following PD-L1 blockade (data not shown). Therefore, in our model, humoral disruption was immutable to blockade of the PD-1 pathway suggesting a more direct role of IFN-I on B cell function.

### IFNAR Signaling Is Essential for LCMV-Mediated Humoral Immune Response Disruption

**Figure 4:**
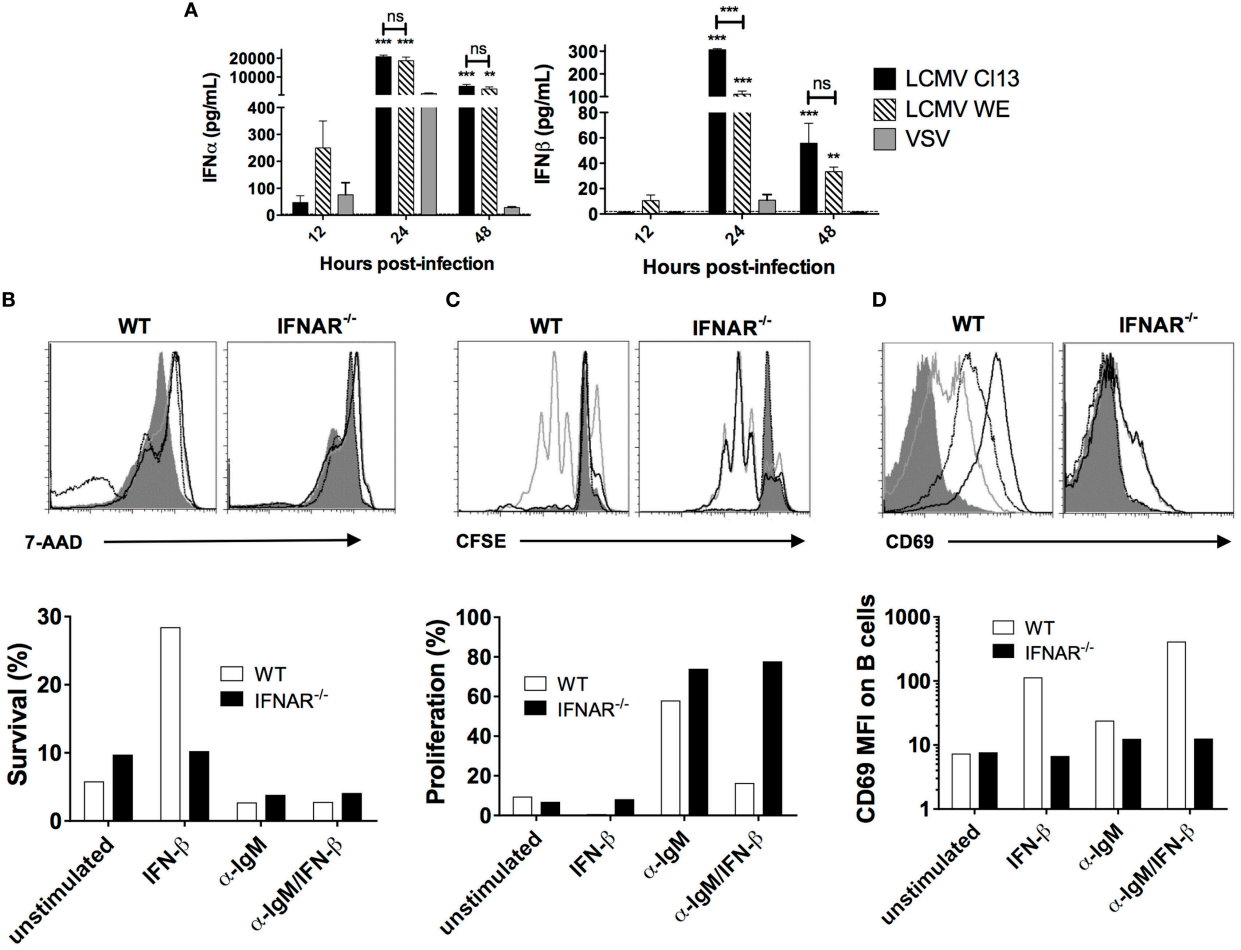
**IFN-I acts in competition with BCR signaling to induce B cell survival and proliferation**. **(A)** Serum level of IFN-I in B6 mice (*n* = 4) infected with LCMV Cl13 (black), LCMV WE (hatched), or VSV (gray) measured using ELISA. Statistical analysis was performed by *T*-test between LCMV and VSV-infected groups. ***p < 0.001. **(B–D)** 2 × 10^6^ B cells from WT B6 (white bars) or IFNAR^−/−^ (black bars) mice were cultured for 7.5 h (for proliferation and CD69 expression) or 4 days (for survival) without *in vitro* stimulation (solid dark gray), or with α-IgM (light gray line), IFN-β (black dotted line), or both (black line). **(B)** 7-AAD exclusion, **(C)** CFSE dilution, and **(D)** CD69 expression were measured by flow cytometry. Representative of two independent experiments.

Previous studies have demonstrated a rapid and robust increase in LCMV-induced IFN-I levels in the serum (16, 17), which, as illustrated in Figure 4A, was also observed in our LCMV cohorts as well as in VSV-infected mice albeit to a much lesser extent and for a shorter duration. Next, we performed *in vitro* stimulation of B cells with IFN-β in the presence or absence of BCR signaling to evaluate any modifications in survival and proliferation. Here, B cell samples were harvested from both wild-type B6 and IFNAR^−/−^ mice to determine specific action by IFN-I. As shown in Figure 4B and consistent with a previous study (24), addition of IFN-β in the WT B cell culture sustained B cell survival by fivefold after 4 days, whereas B cells from IFNAR^−/−^ mice did not respond to IFN-β, as expected. Of note, IFN-I stimulation in this setting is likely equivalent to acute infection and our results are in agreement with the impact of IFN-I on B cells as shown in previous reports (23, 25, 27–29). Interestingly, upon stimulation through the BCR, survival of cultured B cells was diminished regardless of addition of IFN-β. Furthermore, while BCR stimulation increased B cell proliferation, the addition of IFN-β completely abrogated the BCR-dependent increase in proliferation (Figure 4C). We also measured B cell activation by evaluating expression of the activation marker CD69 upon which we found that IFN-β increases B cell activation independently of BCR stimulation (Figure 4D). Given the enhancement of survival independent and antagonistically to BCR signaling, these results suggest that IFN-I signaling could potentiate the increase of non-specific B cells while impairing the development of antigen-specific B cell responses.

**Figure 5:**
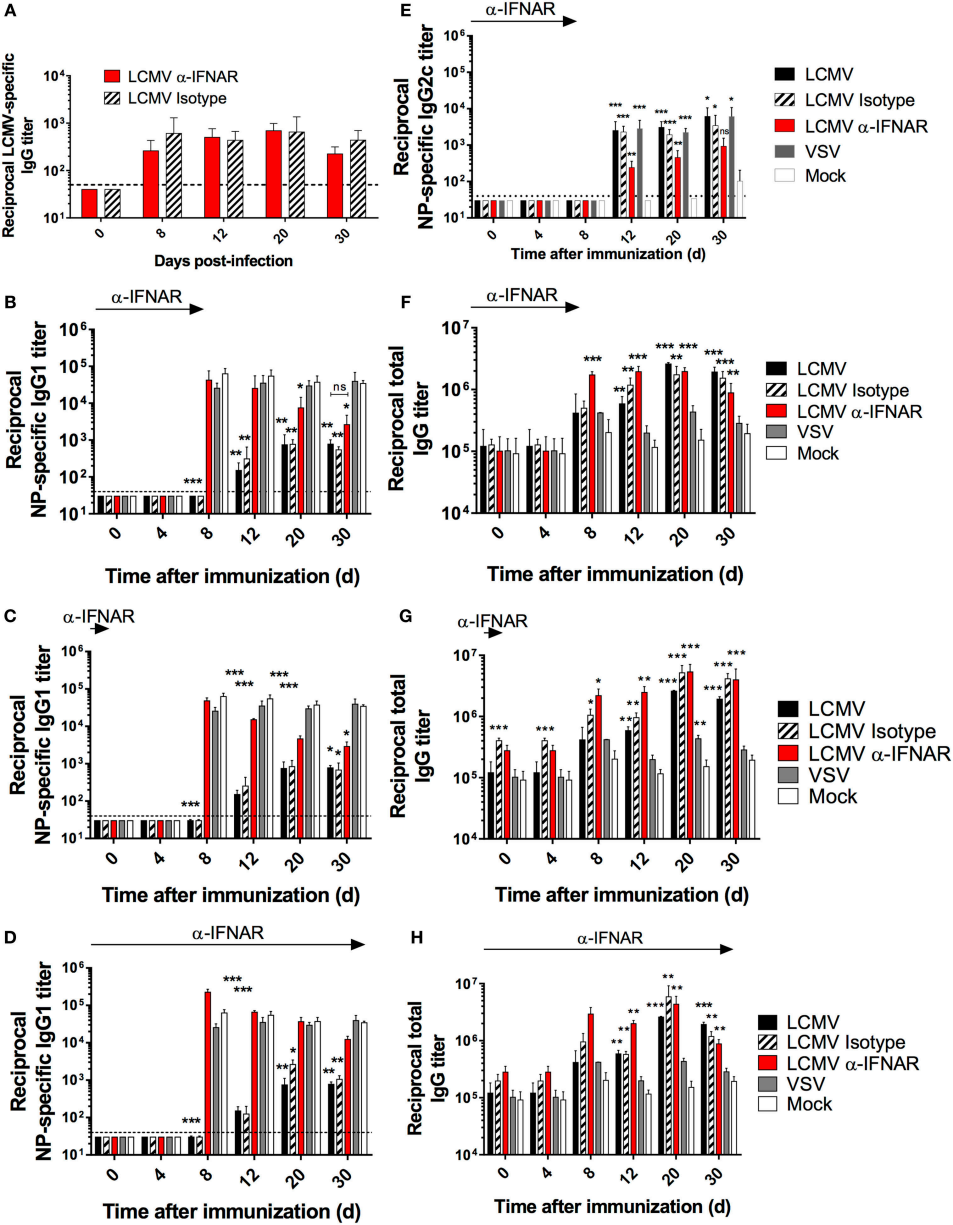
**IFNAR blockade restores NP-specific IgG1 responses during LCMV infection**. B6 mice (four per group) were treated with α-IFNAR Ab (red), isotype control Ab (hatched), or left untreated (black) and were infected the next day with LCMV Cl13, VSV (gray), or mock infected (white). Mice were immunized the same day with an i.p. injection of NP_53_-CGG in alum. **(A,B,E,F)** IFNAR blockade was conducted every second day until d8. In another series of experiments, anti-IFNAR treatment was stopped after either one injection (d-1) **(C,G)** or 11 injections every third day (d30) **(D,H)**. **(A)** LCMV nucleoprotein-specific IgG titers, **(B–D)** NP-specific IgG1, **(E)** NP-specific IgG2c, **(F–H)** total IgG titers monitored by ELISA. Statistical analysis was performed by individual *T*-tests between experimental groups and the mock-infected group. *p < 0.05, **p < 0.01, and ***p < 0.001. The dotted line represents detection threshold. Representative of two independent experiments.

To directly evaluate the effect of IFN-I signaling on the humoral response in our *in vivo* model, we performed LCMV Cl13 infection and co-immunization with NP in mice that were treated with either IFNAR blocking or isotype control Abs. Although IFNAR blockade prior to LCMV Cl13 infection has been shown to enhance viral clearance in a CD4 T cell-dependent manner (16, 17), its impact on the Ab response during the progression of a chronic infection has not been thoroughly assessed. As depicted in Figure 5A, LCMV-specific-binding Ab responses were not significantly affected by IFNAR blockade as observed in previous reports (16, 17). Remarkably, however, NP-specific serum IgG1 titers were restored to levels present in VSV- or mock-infected animals upon IFNAR blockade (Figure 5B). In addition, restoration of the anti-NP IgG1 response was observed following either: a single anti-IFNAR administration conducted on d-1 prior to the infection/immunization (Figure 5C) or a series of 11 anti-IFNAR treatments conducted every third day until d30 postinfection/immunization (Figure 5D). Despite this result, it is important to note that the effect of the short-term Ab treatment regimens seemingly waned over time (Figures 5B,C). IFN-I has been shown to induce CSR primarily toward an IgG2a/c subtype (27). To ascertain that the LCMV-associated depletion of NP-IgG1 responses was not solely due to the skew toward NP-specific IgG2c responses, we assessed whether the recovery of NP-specific IgG1, upon IFNAR blockade, was inversely related to IgG2c titers in our experimental cohorts. As shown in Figure 5E, although low-level NP-specific IgG2c titers were detected starting on d12 following immunization in LCMV-infected mice compared to the mock-infected group, similar levels were also observed in the VSV-infected group indicating a general effect driven by viral infection. Expectedly, IFNAR blockade reduced NP-specific IgG2c titers in LCMV-infected mice denoting a role for IFN-I in the observed CSR to IgG2c. These results indicate that, the IFN-I response generally elicited during all viral infection induces CSR to IgG2c of some antigen-specific B cells, and this effect is unlikely limited to LCMV infection or more broadly, persistent infections; thus, neither the diminished NP-specific IgG1 Ab response observed during LCMV infection nor its recovery upon IFNAR blockade is accounted for by a skewing toward or away from IgG2c responses. Altogether, these results affirm that the suppressive effect observed on the NP-specific IgG1 response during LCMV infection is dependent on IFN-I signaling and independent of CSR to IgG2c. Finally, consistent with a previous report that showed the induction of HGG in IFNAR^−/−^ mice following LCMV infection (38), HGG was unchanged by any of the IFNAR blockade regimen (Figures 5F–H).

**Figure 6:**
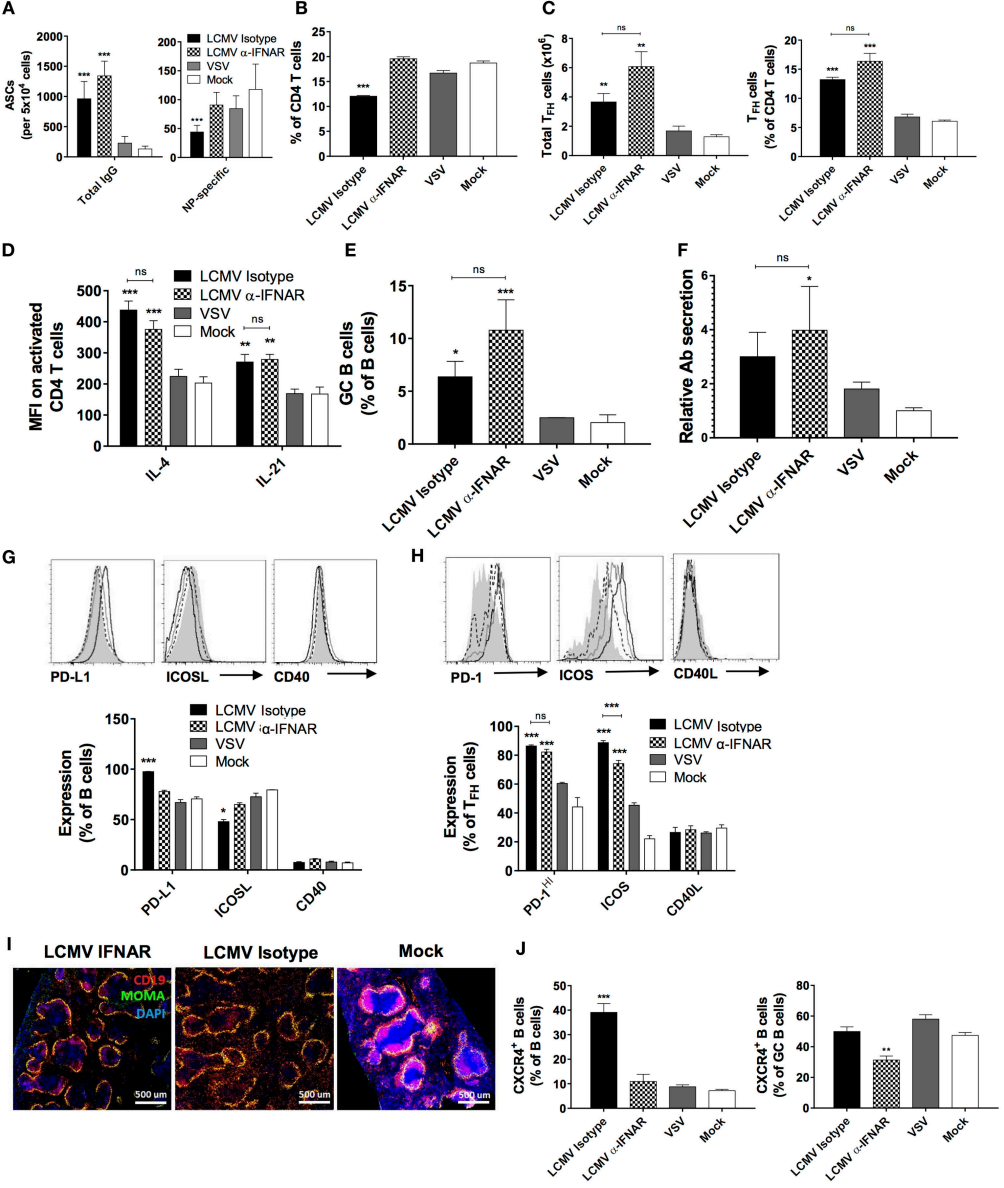
**IFNAR blockade partially restores TD B cell responses and splenic follicular structures**. B6 mice (four per group) were treated with α-IFNAR Ab (checkered) or isotype control Ab (black) and were infected the next day with LCMV Cl13, VSV (gray), or mock infected (white). Mice were immunized the day of the infection with an i.p. injection of NP_53_-CGG in alum. IFNAR blockade was conducted every second day until analysis on d8. **(A)** Number of total (left panel) and NP-specific IgG-secreting (right panel) cells detected by ELISPOT. **(B)** Proportion of CD4 T cells among total lymphocytes and **(C)** total numbers (left panel) and proportions (right panel) of T_FH_ cells among total CD4 T cells as determined by flow cytometry. **(D)** Intracellular IL-4 and IL-21 levels in T_FH_ cells and **(E)** proportion of splenic GC B cells determined by flow cytometry. **(F)** Secretory capacity of ASCs as determined by measuring the quantity of secreted Abs produced by 10^5^ splenocytes. **(G)** Proportion of B cells expressing PD-L1, ICOSL, and CD40. **(H)** Proportion of T_FH_ cells expressing PD-1, ICOS, and CD40L. **(I)** Splenic follicular structures visualized using immunofluorescent staining of CD19 (red), MOMA-1 (green), and DAPI (blue) in tissue sections. **(J)** Proportion of CXCR4^+^ B cells among total (left panel) and GC B cells (right panel) determined by flow cytometry. Statistical analysis was performed by individual *T*-tests between experimental groups and the mock-infected group and between isotype and IFNAR blocking Ab-treated groups when indicated with brackets. *p < 0.05, **p < 0.01, and ***p < 0.001. **(A,J)** Representative of three independent experiments. **(B–I)** Representative of two independent experiments.

We next evaluated the impact of IFNAR blockade on the TD B cell response during LCMV infection. Consistent with the normalized IgG1 response against NP, the number of NP-specific IgG-secreting cells returned to that found in VSV- or mock-infected animals (Figure 6A, right panel) upon IFNAR blockade while total IgG-secreting cells remained elevated (Figure 6A, left panel), in agreement with sustained HGG. Surprisingly, although IFNAR blockade led to the restoration of total splenic CD4+ T cell proportions (Figure 6B), the increase in T_FH_ observed following LCMV infection remained unchanged (Figure 6C). Moreover, the treatment bore limited impact on IL-4 and IL-21 expression in CD4 T cells (Figure 6D), GC B cell proportions (Figure 6E), and relative Ab secretion by ASCs (Figure 6F). However, relative to the isotype control, anti-IFNAR treatment resulted in moderate modulation in the expression levels of PD-L1, ICOSL, and ICOS (Figures 6G,H). As observed, T_FH_ and GC B cell populations remained elevated upon IFNAR blockade despite the recovery of NP-specific Abs. Next, we sought to determine whether the structure of B cell follicles was restored upon IFNAR blockade similar to the rescue of lymphoid architecture as described in previous reports (16, 17, 20). Here, we observed that only incomplete recovery of the B cell follicle structure occurred suggesting that LCMV infection induces disruption of B cell localization in a partially IFNAR-independent manner (Figure 6I). Based on the role of CXCR4 in the trafficking of B cells in lymphoid follicles and resultantly their structure (44), we examined its expression level upon IFNAR blockade. As shown in Figure 6J, left panel, we observed a remarkable elevation in CXCR4 expression in total B cells, which was restored to base levels upon IFNAR blockade. Surprisingly however, this increase in CXCR4 expression levels was not observed in the GC compartment in the LCMV-infected group compared to VSV- or mock-infected animals (Figure 6J, right panel). Nonetheless, IFNAR blockade also significantly reduced CXCR4 expression in GC B cells. These results illustrate a significant role played by IFN-I produced during LCMV infection in altering CXCR4 expression and consequently the trafficking and localization of B cells outside of follicular structures. This interplay suggests a mechanistic outline by which IFN-I modulates the humoral immune response.

### B Cell-Intrinsic IFN-I Signaling Directly Disrupts the Antigen-Specific Humoral Response

**Figure 7:**
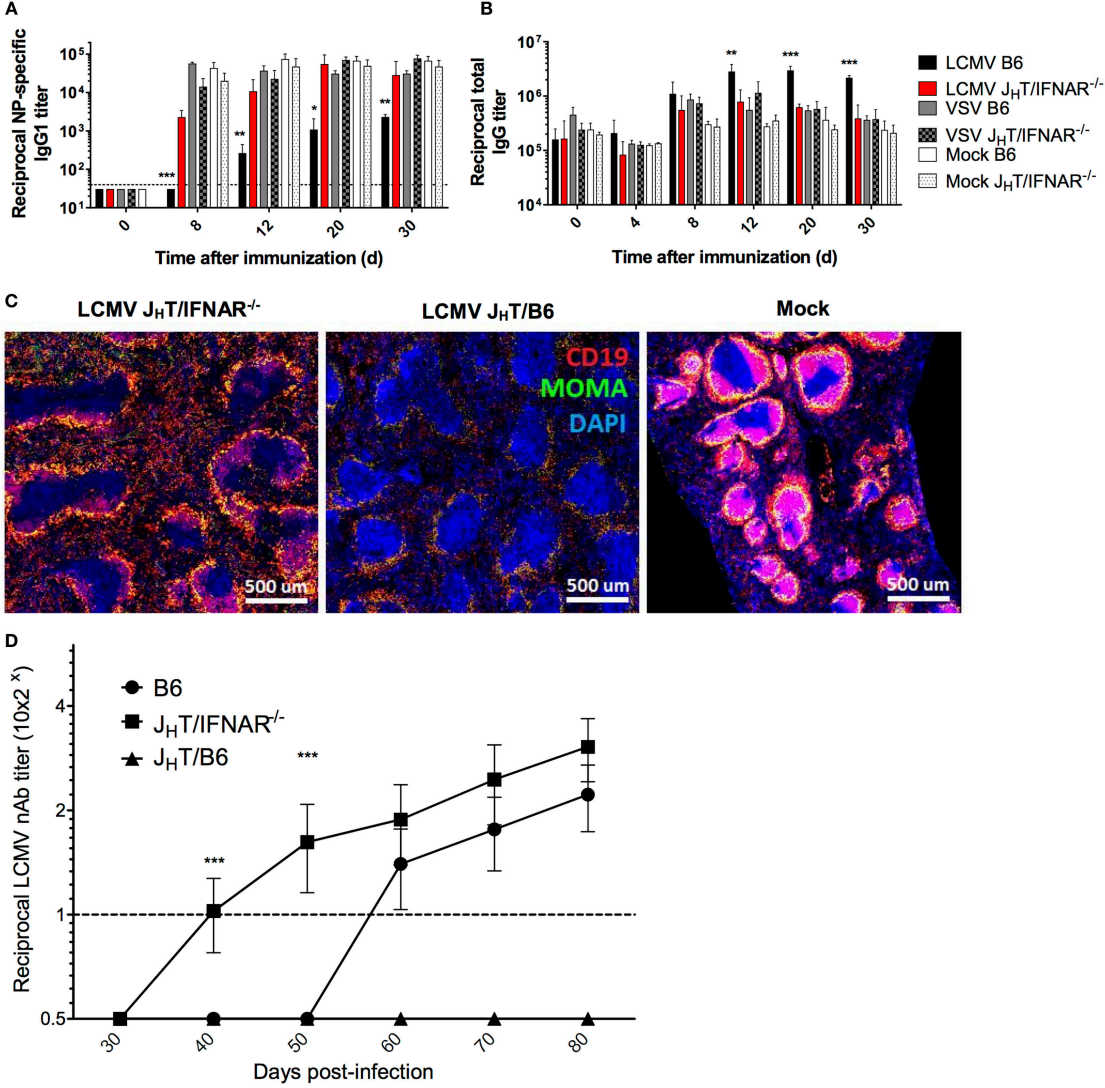
**B cell-specific disruption of IFNAR restores NP-specific Ab responses and accelerates the development of LCMV nAbs**. **(A–C)** B6, J_H_T/IFNAR^−/−^, or J_H_T/B6 mice (four per group) were infected with LCMV Cl13 (black for B6; red for J_H_T/IFNAR^−/−^), VSV (gray), or were mock infected (white). Plain bars represent B6 mice and checkered bars J_H_T/IFNAR^−/−^ B cell bone marrow chimeric mice. **(A)** NP-specific IgG1 and **(B)** total IgG responses monitored using ELISA on d8 following infection/NP_53_-CGG immunization. **(C)** Splenic follicular structures visualized using immunofluorescent staining of CD19 (red), MOMA-1 (green), and DAPI (blue) on spleen sections obtained on d8 postinfection. **(D)** Neutralization assay showing accelerated nAb responses in J_H_T/IFNAR^−/−^ chimeric mice upon infection with LCMV Cl13. **(A–C)** Representative of two independent experiments. **(D)** Compilation of three independent experiments (B6 mice, *n* = 21; J_H_T/IFNAR^−/−^ mice, *n* = 25; J_H_T/B6 mice, *n* = 8). Statistical analysis was performed by individual *T*-tests between experimental groups and the mock-infected group for **(A–C)** and one-way ANOVA for **(D)**. *p < 0.05, **p < 0.01, and ***p < 0.001. The dotted line represents detection threshold.

Our data so far have suggested an effect of IFN-I on B cells. Therefore, to determine the role of B cell-intrinsic IFN-I signaling on the impaired humoral response observed during LCMV infection, we developed a chimeric model by reconstituting irradiated B6 mice with a mix of bone marrow cells from B cell-deficient (J_H_T) mice (45) and IFNAR^−/−^ mice. As a result, we obtained chimeras in which only the B cells are deficient in IFN-I signaling. Upon LCMV Cl13 infection and NP-CGG immunization of J_H_T/IFNAR^−/−^ chimeras, we observed restoration of the NP-specific response to levels observed in the VSV- or mock-infected B6 mice (Figure 7A), which was consistent with the IFNAR blockade experiment. Interestingly, whereas IFNAR blockade did not ameliorate HGG, the levels of total serum IgG in LCMV Cl13-infected J_H_T/IFNAR^−/−^ chimeras were significantly lower than those in WT animals and normal relative to VSV and mock-infected mice (Figure 7B). Yet, despite the normalization of humoral immune responses, J_H_T/IFNAR^−/−^ mice still exhibited only partial rescue of the splenic marginal-zone and B cell follicle structures (Figure 7C). Perhaps the most significant impact observed by the absence of B cell-specific IFNAR signaling was the accelerated emergence of nAbs in the chimeras compared to both J_H_T/B6 chimeras, used as controls to account for any changes driven by irradiation and reconstitution and more importantly, WT B6 mice (Figure 7D). The fact that J_H_T/B6 chimeras did not produce any detectable LCMV nAbs suggests that only partial reconstitution of the humoral response was achieved in the chimeric system. This result further underscores the significance of the accelerated nAb response observed in the J_H_T/IFNAR^−/−^ chimeras. Collectively, these data illustrate a potent effect borne by IFN-I signaling on B cells, which upon negation results in a normal NP-specific humoral response as well as enhanced induction of LCMV nAbs.

## Discussion

The immune response in the LCMV infection model has been classically defined as cell mediated (46) whereas the role of the humoral immune response has only been considered relevant in the context of reinfection (47). Although the absence of an initial robust nAb response has been primarily attributed to antiviral escape mechanisms (32, 48), the immunological processes that drive the disruption of humoral immunity during persistent infection are yet to be elucidated.

In this report, we reveal that emergence of humoral dysfunction during LCMV infection occurs in an IFN-I-dependent manner in which antigen specificity in the TD immune response was impaired; this effect was notably more evident in LCMV Cl13- relative to WE-infected animals. To gain deeper insight into the nature of immunological impairment, we tracked the immune response against NP rather than the actual viral antigens in different experiments to distinguish viral specific effects from global immune responses. Collectively, the presence of humoral disruption observed with both LCMV strains illustrates that pathogenic mechanisms present in the acute phase of infection as well as sustenance of viral burden in the face of prolonged LCMV Cl13 infection both play a role in the observed perturbations. However, this impairment is not a universal consequence of any viral infection or of IFN-I production *per se* as infection with VSV, which also promotes IFN-I production, albeit to a lesser extent than LCMV, does not lead to a disrupted humoral response; as such, LCMV-specific factors also likely play a role in the muted Ab response. Whereas blockade of IFN-I signaling using anti-IFNAR Abs reestablished the anti-NP response, HGG remained present consistent with a previous report in IFNAR^−/−^ (38) and the expansion of the T_FH_ and total IgG-secreting cell populations was still observed. Furthermore, the expression of costimulatory molecules between B and T cells was partially restored to levels intermediate between isotype treated and VSV-infected controls. In addition, treatment with anti-PD-1/PD-L1, which was conducted prior to infection and co-immunization, bore no impact on LCMV-mediated dysregulation. These findings along with the observed effect of IFN-I on B cell survival and proliferation *in vitro* suggested that LCMV-induced IFN-I acted directly on B cells. The significance of this interaction was demonstrated using J_H_T/IFNAR^−/−^ chimeric mice in which only B cells carried the receptor knockout. Remarkably, the absence of IFN signaling on B cells restored normal humoral function with NP-specific Ab titers similar to those observed in VSV- or mock-infected mice. Moreover, B cell-specific IFNAR ablation normalized HGG consistent with a recent report in the Leishmania infection model (49). In light of the incapacity of IFNAR blockade to limit HGG, this suggests that IFN-I signaling on cells other than B cells also contributes to regulating HGG development although the exact nature of this contribution remains to be defined. Altogether, these results revealed the role of IFN-I signaling on B cells in impairing Ag-specific responses albeit possibly indirectly through the abnormal expansion of non-specific B cells.

Along with the recovery of the antigen-specific humoral response, we observed a reduction in the extent of destruction to splenic follicular architecture upon both the blockade and B cell-specific knockout of IFNAR. Similar to the recovery of the general splenic tissue organization observed in previous studies (16, 17), we surmise that this occurs in part due to preservation of cellular trafficking and localization in the splenic microstructure. Shuttling back and forth between the light zone (LZ) and dark zone (DZ) of the GC is actuated by differential expression of chemokine receptors such as CXCR4 and CXCR5 (50, 51). Upon analysis of CXCR4 expression on B cells, we observed that the LCMV-associated increase in total B cells was completely reversed by anti-IFNAR Ab administration. However, since CXCR4 expression on GC B cells was unchanged upon LCMV infection, we surmise that the overall increased expression of the chemokine receptor upon infection most likely occurred in extrafollicular B cells. It is important to note that this change to cellular trafficking and localization likely represents only a fraction of similar such changes that alter splenic structure during LCMV infection.

**Figure 8:**
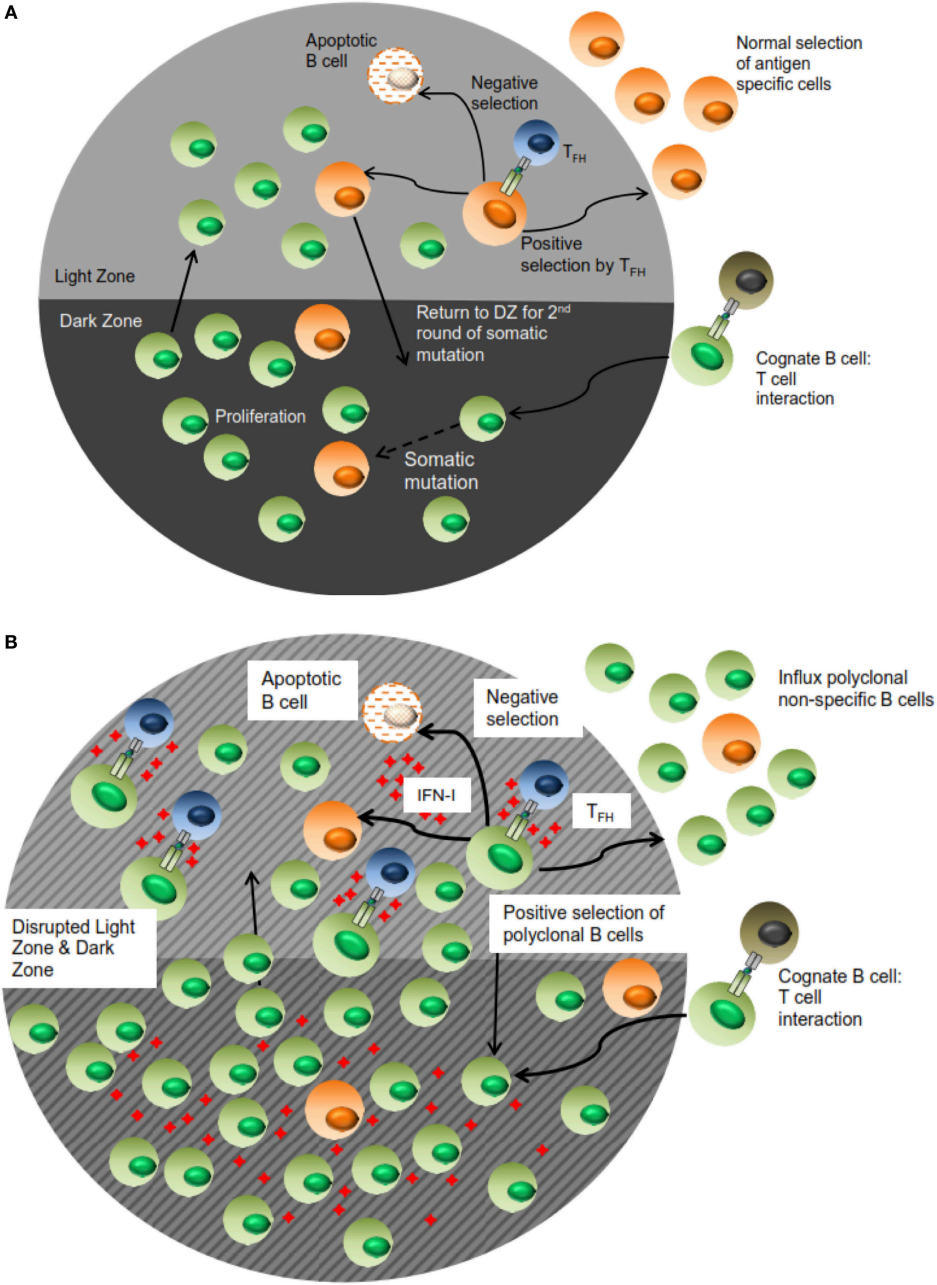
**Proposed schematic of B cell selection during a normal versus impaired humoral immune response during LCMV infection**. In **(A)**, antigen specificity of the resultant Ab response is facilitated by effective selection of B lymphocytes in the LZ of the GC response. Some of these cells migrate to the periphery as effector cells while others cycle back into the DZ for proliferation in an iterative process. During LCMV and more generally, other chronic infections **(B)**, the high concentration of IFN-I along with sustained elicitation and T_FH_ expansion triggers selection of non-specific B cells in the context of a disrupted follicular architecture; as such, antigen-specific effector cells and consequently, Ab responses, are “drowned” out by the influx of non-specific cells. This figure only represents a snapshot of the process in which the immune response is further honed in **(A)**, whereas the dilution and loss of specificity in **(B)** is exacerbated in subsequent rounds.

In addition, these changes to B cell trafficking also suggest altered positive selection thresholds of effector cells. This possibility is further advanced by the increase in GC B cell numbers as well as the expansion of the non-specific ASC population_._ Along with the observation of T_FH_ increase in LCMV-infected animals, we deduce that the positive selection process in which antigen-specific B cell clones compete for survival signals from T_FH_ is dysregulated. In this setting, the threshold for positive selection is lowered based on the aberrant expansion of T_FH_ cells. As a result, B cells exhibiting lower antigen specificity or non-specificity, which in the normal functional setting would not be selected, receive survival signals. Consequently, this expansion of non-specific B cells diminishes the likelihood of cognate T_FH_–B cell interactions taking place in a productive manner, which leads to an impairment of specific Ab responses (see Figure 8 for proposed model). However, the affinity maturation process is still intact despite this disruption based on the stability of the high affinity NP_4_-BSA Ab titers in all the infection groups. Thus, the positive selection mechanism is functional yet stunned by the influx of non-specific B cell clones. Most significantly, the accelerated LCMV nAb response observed in the J_H_T/IFNAR^−/−^ chimeras relative to WT B6 and J_H_T/B6 can also be accounted for by such a mechanism. In this setting, the WT response features increased activation of non-specific B cell clones. Consequently, the response to neutralizing epitopes, which are immunorecessive, is further diminished by the effects of IFN-I signaling. This is a significant characterization given that the elicitation of nAbs and moreover, broadly nAbs, is also delayed in chronic infection settings such as HIV and HCV whose immunological profiles closely mirror those observed in LCMV.

Another important aspect of human persistent virus infection is poor vaccination responses in infected patients. Using NP co-immunization, our model recapitulates the diminished response against a secondary antigen and elucidates the role played by IFN-I-mediated signaling in the chronic infection setting. Other studies have also shown that poor responses against vaccines are correlated with an increased PD-1:PD-L1 profile in HIV and HCV infected patients (10, 52).

In conclusion, the lack of an effective humoral immune response during LCMV infection is driven by general modulation to the humoral immune microenvironment by IFN-I. This modulation is limited to the TD response and is established early following infection; as well, blockade of IFN-I-mediated effects allows the immune response to proceed normally. Altogether, there are many aspects of the immune response to consider in their entirety, but the significance of IFN-I signaling offers an ideal anchor for future analysis and provides a therapeutic target against persistent viral infections to potentially boost Ab responses as well as limit immunopathogenesis.

## Materials and Methods

### Ethics Statement

All animal experiments were reviewed and approved by the Institut national de la recherche scientifique (INRS) animal care committee under protocol 1302-05 and in accordance with AAALAC and Canadian Council on Animal Care (CCAC) guidelines.

### Mice, Infections, and Immunizations

Six- to eight-week-old B6 female mice were purchased from Charles River Laboratories (St-Constant, QC, Canada). J_H_T mice were obtained from Rolf M. Zinkernagel, Zurich, Switzerland. IFNAR^−/−^ mice on a B6 background were obtained from Ulrich Kalinke, Hannover, Germany. All mice were maintained at the INRS animal facility until use.

To generate mixed bone marrow chimeric mice, bone marrow cells from 8- to 12-week-old B6, J_H_T, and IFNAR^−/−^ mice were mixed at a ratio of 4:1 (J_H_T/IFNAR^−/−^ or J_H_T/B6) and injected intravenously (i.v.) into lethally irradiated (2 × 600 rads) B6 mice. Mice were left untouched for ≥5 weeks to allow for immune reconstitution as determined by flow cytometry.

All viral strains used in this paper were obtained from Rolf M. Zinkernagel, Zurich, Switzerland. Infections with LCMV Cl13, LCMV WE, or VSV Indiana were carried out by i.v. injection with 2 × 10^6^ pfu of virus or with culture media alone (mock).

For T cell-dependent Ab response characterization, mice were immunized intraperitoneally (i.p.) with 50 μg alum-precipitated 4-Hydroxy-3-nitrophenylacetic hapten conjugated to CGG (NP-CGG; Biosearch Technologies, Petaluma, CA, USA), at a ratio of 53:1, or with precipitated alum alone as a control. For T cell-independent Ab response characterization, mice were immunized i.p. with 5 μg 4-hydroxy-3-nitrophenylacetic hapten conjugated to AminoEthylCarboxyMethyl-FICOLL (NP-FICOLL; Biosearch Technologies, Petaluma, CA, USA) in PBS, at a ratio of 40:1, or with PBS alone as a control. All mice were maintained under specific pathogen-free conditions and immunizations or adoptive transfers were carried out at 6–10 weeks of age.

Blocking Abs were used to block PD-1 and IFNAR signaling. For PD-1 blockade, 250 μg of blocking Ab or isotype control (BioXcell, West Lebanon, NH, USA) were injected i.p. every 3 days starting 1 day before infection and continuing throughout the whole experiment. For IFNAR blockade, 500 μg of blocking Ab or isotype control (Leinco Technologies) were injected i.v. every 2–3 days starting 1 day before infection and stopping at various time points as indicated.

### B Cell *In Vitro* Stimulation and CFSE Staining

Splenic B cells were isolated using the EasySepTM Mouse B Cell Enrichment Kit (Stemcell Technologies, Vancouver, BC, Canada) according to the manufacturer’s instructions. B cells were resuspended to 2 × 10^7^ cells/mL in PBS containing 2% FBS and CFSE (Fisher, Waltham, MA, USA) was added to a final concentration of 1 μM. Samples were incubated for 5 min at room temperature and staining was quenched by addition of an equal amount of FBS. Cells were washed with PBS/2% FBS and resuspended in RPMI-1640 medium supplemented with 10% FBS, 2 mM glutamine, 100 U/mL penicillin, 100 μg/mL streptomycin, and 55 μM 2-mercaptoethanol. The 3 × 10^6^ cells/well were seeded in 24-well plates and stimulated with either 10 μg/mL goat anti-mouse IgM (Jackson Immunoresearch, West Grove, PA, USA) and 500 U/mL mouse IFN-β (Quansys Biosciences, Logan, UT, USA) or respective combinations and cultured (37°C, 5% CO2) for either 7.5 h or 4 days.

### Antibodies and Detection Reagents

The following staining reagents were used: anti-CD3 (145-2C11), anti-CD4 (H129.19), anti-CD62L (MEL-14), anti-CD44 (IM7), anti-CD45R (RA3-6B2), anti-PD-1 (29F.1A12), anti-PD-L1 (10F.9G2), anti-ICOSL (HK5.3), anti-CD40 (HM40-3), anti-CD40L (MR1), anti-CXCR4 (L276F12), and anti-IL-4 (11b11) were from Biolegend (San Diego, CA, USA); anti-CD19 (eBio1D3), anti-ICOS (7E.17G9), anti-IL-21 (mhalx21), anti-CD69 (H1.2F3), and anti-GL7 (GL-7) were from eBiosciences (San Diego, CA, USA); anti-Bcl-6 (K112-91), anti-CXCR5 (2G8), and anti-Fas (Jo2) were from BD Biosciences (Franklin Lakes, NJ, USA). PNA (B-1075; Vector Laboratories, Burlington, ON, Canada) was used for staining germinal center B cells and MOMA-1 (Abcam, Cambridge, UK) for staining MZ macrophages.

### Flow Cytometry

Single-cell suspensions of splenocytes were stained on ice with the appropriate Abs in PBS containing 1% (v/v) bovine serum albumin (BSA, Fisher, Waltham, MA, USA) and 0.1% (w/v) sodium azide (GE Healthcare, Mississauga, ON, Canada). Non-specific staining was blocked by 2.4G2 Ab against CD16/32 [Biolegend; prepared as previously described (53)]. Samples were fixed with 1% (v/v) paraformaldehyde and analyzed on a BD LSR II Fortessa (BD Bioscience). For intracellular cytokine staining, splenocytes were stimulated *ex vivo* for 5 h with phorbol 12-myristate 13-acetate (PMA, Sigma-Aldrich, Saint-Louis, MI, USA) (100 ng/mL), ionomycin (Sigma-Aldrich) (1 μM), and Brefeldin A (Sigma-Aldrich) (10 μg/mL). Samples were fixed and permeabilized prior to incubation with Ab in permeabilization buffer (eBiosciences). Dead cells were excluded based on positive staining with 7-Amino-Actinomycin D (7-AAD) (BD Pharmingen), and doublets gated out using FSA-A/SSC-A. Data were analyzed with FlowJo software (TreeStar, Ashland, OR, USA).

### Quantitative RT-PCR

CD4^+^ T cells were magnetically selected using the EasySep system (StemCell, Vancouver, BC, Canada), frozen, and conserved at −80°C. Total RNA was isolated using the RNeasy Mini kit (Qiagen, Hilden, Germany) and quantified by Nanodrop ND-1000 (Fisher, Waltham, MA, USA). cDNA was synthesized from total RNA using the Superscript III First-Strand Synthesis SuperMix (Applied Biosystems) and frozen at −80°C until use. Gene expression was determined by quantitative PCR using TaqMan Gene expression System (Life Technologies, Burlington, ON, Canada) on a Rotor-gene 6000 system (Corbett, Concorde, NSW, Australia). Standard commercial TaqMan probes were used for IL-4, IL-21 and BAFF (Life Technologies). Samples were normalized to GAPDH and represented as fold change over mock-infected mice using the ΔΔCT method (54).

### ELISA and ELISPOT Assay

For NP-specific serum Ig detection, 96-well plates (Mabtech, Cincinnati, OH, USA) were coated overnight at 4°C with 0.1 μg of NP-BSA conjugated at a ratio of NP to BSA ranging from 4:1 to 26:1. For total serum IgG detection, microtiter plates were coated overnight at 4°C with unlabeled anti-IgG (Jackson ImmunoResearch, West Grove, PA, USA) at 2 μg/mL. Non-specific binding was blocked with 10% (v/v) fetal bovine serum and 0.2% (v/v) Tween 20 in PBS. Horseradish peroxidase (HRP)-conjugated secondary Abs: anti-IgM, anti-IgG1, anti-IgG2c, anti-IgG3, and anti-IgG (total IgG) (Jackson ImmunoResearch, West Grove, PA, USA) were detected with 0.04% (w/v) o-phenylenediamine and 0.8% H_2_O_2_ (v/v) in citrate buffer. Serum BAFF detection was done by coating 96-well plates overnight at 4°C with 2 μg of anti-BAFF Ab (R&D Systems, Minneapolis, MN, USA) in carbonate buffer. Non-specific binding was blocked with 1% (v/v) BSA in PBS. HRP-conjugated anti-BAFF secondary Ab (R&D Systems) was detected with 0.04% (w/v) o-phenylenediamine and 0.8% H_2_O_2_ (v/v) in citrate buffer. Serum IFN-α and -β detection was done using Verikine kits (PBL Assay Science, Piscataway Township, NJ, USA) in conditions recommended by the company. LCMV nucleoprotein-specific IgG serum Abs were determined by ELISA using plates coated with purified recombinant LCMV nucleoprotein-GST as previously described (55).

For ASC determination by ELISPOT, a mouse IgG ELISPOT^Plus^ kit (Mabtech, Nacka Strand, Sweden) was used. Briefly, 96-well nitrocellulose plates (Millipore, Etobicoke, ON, Canada) were coated overnight at 4°C with anti-IgG antibody. Non-specific binding was blocked with 5% (v/v) FBS in PBS. Cell suspensions obtained from spleens were added to wells (10^4^ cells for detection of IgG and 10^5^ for NP) in duplicate and incubated overnight at 37°C in a 5% CO_2_ atmosphere. B cell spots were developed by sequential washes with PBS, addition of biotinylated anti-IgG or NP-BSA-Biotin, washes with PBS, addition of streptavidin-alkaline phosphatase, washes with PBS and addition of 5-Bromo-4-chloro-3-indolyl phosphate substrate for 15 min. Spots were counted using an AID Elispot Reader (Autoimmun Diagnostika GmbH, Strasberg, Germany).

### Immunochemistry

Freshly harvested spleens were flash frozen in OCT (Electron Microsopy Sciences, Hartfield, PA, USA) in liquid nitrogen, stored at −80°C and processed for sets of 10-μm section sizes with a cryostat (Microm HM 525; GMI, Ramsey, MN, USA). Tissue sections were fixed on slides in 75% acetone and 25% ethanol (v/v) for 5 min and incubated with primary reagent in PBS for 1 h at room temperature. The following primary reagents were used: anti-mouse CD19-PE (RA3-6B2, eBiosciences, 1:10000) and anti-mouse MOMA-1-FITC (Abcam, Toronto, ON, Canada, 1:200). Tissue sections were then washed in PBS and incubated with a secondary Alexa 488 anti-FITC (Life Technologies) to amplify FITC signal or strep-A488 if needed. Tissue sections were then mounted with Prolong (Life Technologies), dried overnight, and observed using a LSM780 confocal microscope (Carl Zeiss, Oberkochen, Germany).

### Ab Secretion Quantification

Freshly prepared splenocyte suspensions were plated in triplicates in 96-well flat bottom plates at 10^5^ cells per well in culture medium containing DMEM, 10% FBS, 1% streptavidin/penicillin, 1% l-glutamine, 1% sodium pyruvate, and β-mercaptoethanol. Cells were incubated for 48 h at 37°C and culture media was harvested to quantify Ab by ELISA. This quantitation was compared to the numeration of ASCs obtained using ELISPOT to define an individual secretion per ASC cell.

### LCMV nAb Quantification

Lymphocytic choriomeningitis virus nAbs were quantified as described previously (56). In brief, serial 2-fold dilutions of 10-fold prediluted sera were incubated with LCMV for 90 min at 37°C in 96-well plates. MC57G mouse fibroblasts were added and incubated for 1 h to allow cells to settle and be infected by non-neutralized virus; cells were then overlaid with 1% methylcellulose in MEM. After 48 h, cell monolayers were fixed with 4% formalin and infectious foci were detected by intracellular LCMV staining of infected cells with rat anti-LCMV mAb VL-4.

### Statistical Analysis

Data were analyzed using Prism 6 (GraphPad Software, Inc.). Statistical significance was assessed as indicated using unpaired two-sided *T*-test, or a one-way ANOVA with Tukey’s multiple comparisons test. *p* Value <0.05 was considered significant. *p < 0.05, **p < 0.01, and ***p < 0.001. Data are represented as means ± SD.

## Author Contributions

MD and AM designed and performed experiments, analyzed data, and wrote the paper; BM designed and performed experiments and analyzed data; AG and ET performed experiments; PL helped to analyze data; JF designed parts of the study and analyzed data; AL directed the study, analyzed data and organized, designed, and wrote the paper.

## Conflict of Interest Statement

The authors declare that the research was conducted in the absence of any commercial or financial relationships that could be construed as a potential conflict of interest.

## Supplementary Material

Figure S1 **Kinetics of the LCMV WE-dependent impairment of the NP-specific Ab response**. B6 mice (four per group) were infected with LCMV WE (black), VSV Ind (gray), or mock infected (white). Mice were then immunized with an i.p. injection of NP_53_-CGG in alum following a varying immunization schedule (from d-4 to d30). **(A)** NP-specific IgG1 response in each differentially timed experiment monitored by ELISA. **(B)** ELISA plates were coated with NP_4_-BSA or NP_26_-BSA and high affinity Ab responses were measured as a ratio of Abs binding to NP_4_-BSA versus the total anti-NP IgG1 response binding to NP_26_-BSA in each differentially timed experiment. Statistical analysis was performed by individual *T*-tests between experimental groups and the mock-infected group. **p* < 0.05, ***p* < 0.01, and ****p* < 0.001.

## References

[1] Murira A Lapierre P Lamarre A. Evolution of the humoral response during HCV infection: theories on the origin of broadly neutralizing antibodies and implications for vaccine design. Adv Immunol (2016) 129:55–107. 10.1016/bs.ai.2015.09.004 26791858

[2] Charles ED Green RM Marukian S Talal AH Lake-Bakaar GV Jacobson IM Clonal expansion of immunoglobulin M+CD27+ B cells in HCV-associated mixed cryoglobulinemia. Blood (2008) 111(3):1344–56. 10.1182/blood-2007-07-101717 17942751 PMC2214737

[3] Moir S Ho J Malaspina A Wang W DiPoto AC O’Shea MA Evidence for HIV-associated B cell exhaustion in a dysfunctional memory B cell compartment in HIV-infected viremic individuals. J Exp Med (2008) 205(8):1797–805. 10.1084/jem.20072683 18625747 PMC2525604

[4] De Milito A Nilsson A Titanji K Thorstensson R Reizenstein E Narita M Mechanisms of hypergammaglobulinemia and impaired antigen-specific humoral immunity in HIV-1 infection. Blood (2004) 103(6):2180–6. 10.1182/blood-2003-07-2375 14604962

[5] Racanelli V Frassanito MA Leone P Galiano M De Re V Silvestris F Antibody production and in vitro behavior of CD27-defined B-cell subsets: persistent hepatitis C virus infection changes the rules. J Virol (2006) 80(8):3923–34. 10.1128/JVI.80.8.3923-3934.2006 16571809 PMC1440441

[6] He B Qiao X Klasse PJ Chiu A Chadburn A Knowles DM HIV-1 envelope triggers polyclonal Ig class switch recombination through a CD40-independent mechanism involving BAFF and C-type lectin receptors. J Immunol (2006) 176(7):3931–41. 10.4049/jimmunol.176.7.3931 16547227

[7] Qiao X He B Chiu A Knowles DM Chadburn A Cerutti A. Human immunodeficiency virus 1 Nef suppresses CD40-dependent immunoglobulin class switching in bystander B cells. Nat Immunol (2006) 7(3):302–10. 10.1038/ni1302 16429138

[8] Pallikkuth S Parmigiani A Silva SY George VK Fischl M Pahwa R Impaired peripheral blood T-follicular helper cell function in HIV-infected nonresponders to the 2009 H1N1/09 vaccine. Blood (2012) 120(5):985–93. 10.1182/blood-2011-12-396648 22692510 PMC3412336

[9] Moir S Fauci AS. Insights into B cells and HIV-specific B-cell responses in HIV-infected individuals. Immunol Rev (2013) 254(1):207–24. 10.1111/imr.12067 23772622

[10] Cubas RA Mudd JC Savoye AL Perreau M van Grevenynghe J Metcalf T Inadequate T follicular cell help impairs B cell immunity during HIV infection. Nat Med (2013) 19(4):494–9. 10.1038/nm.3109 23475201 PMC3843317

[11] Feng J Hu X Guo H Sun X Wang J Xu L Patients with chronic hepatitis C express a high percentage of CD4(+)CXCR5(+) T follicular helper cells. J Gastroenterol (2012) 47(9):1048–56. 10.1007/s00535-012-0568-1 22426636

[12] Fahey LM Wilson EB Elsaesser H Fistonich CD McGavern DB Brooks DG. Viral persistence redirects CD4 T cell differentiation toward T follicular helper cells. J Exp Med (2011) 208(5):987–99. 10.1084/jem.20101773 21536743 PMC3092345

[13] Osokine I Snell LM Cunningham CR Yamada DH Wilson EB Elsaesser HJ Type I interferon suppresses de novo virus-specific CD4 Th1 immunity during an established persistent viral infection. Proc Natl Acad Sci U S A (2014) 111(20):7409–14. 10.1073/pnas.1401662111 24799699 PMC4034239

[14] McNab F Mayer-Barber K Sher A Wack A O’Garra A. Type I interferons in infectious disease. Nat Rev Immunol (2015) 15(2):87–103. 10.1038/nri3787 25614319 PMC7162685

[15] Oldstone MB. A Jekyll and Hyde profile: type 1 interferon signaling plays a prominent role in the initiation and maintenance of a persistent virus infection. J Infect Dis (2015) 212(Suppl 1):S31–6. 10.1093/infdis/jiu501 26116728 PMC4574552

[16] Teijaro JR Ng C Lee AM Sullivan BM Sheehan KC Welch M Persistent LCMV infection is controlled by blockade of type I interferon signaling. Science (2013) 340(6129):207–11. 10.1126/science.1235214 23580529 PMC3640797

[17] Wilson EB Yamada DH Elsaesser H Herskovitz J Deng J Cheng G Blockade of chronic type I interferon signaling to control persistent LCMV infection. Science (2013) 340(6129):202–7. 10.1126/science.1235208 23580528 PMC3704950

[18] Barber DL Wherry EJ Masopust D Zhu B Allison JP Sharpe AH Restoring function in exhausted CD8 T cells during chronic viral infection. Nature (2006) 439(7077):682–7. 10.1038/nature04444 16382236

[19] Brooks DG Trifilo MJ Edelmann KH Teyton L McGavern DB Oldstone MB. Interleukin-10 determines viral clearance or persistence in vivo. Nat Med (2006) 12(11):1301–9. 10.1038/nm1492 17041596 PMC2535582

[20] Ng CT Sullivan BM Teijaro JR Lee AM Welch M Rice S Blockade of interferon beta, but not interferon alpha, signaling controls persistent viral infection. Cell Host Microbe (2015) 17(5):653–61. 10.1016/j.chom.2015.04.005 25974304 PMC4432251

[21] Wilson EB Kidani Y Elsaesser H Barnard J Raff L Karp CL Emergence of distinct multiarmed immunoregulatory antigen-presenting cells during persistent viral infection. Cell Host Microbe (2012) 11(5):481–91. 10.1016/j.chom.2012.03.009 22607801 PMC3359873

[22] Coro ES Chang WL Baumgarth N. Type I IFN receptor signals directly stimulate local B cells early following influenza virus infection. J Immunol (2006) 176(7):4343–51. 10.4049/jimmunol.176.7.4343 16547272

[23] Le Bon A Schiavoni G D’Agostino G Gresser I Belardelli F Tough DF. Type I interferons potently enhance humoral immunity and can promote isotype switching by stimulating dendritic cells in vivo. Immunity (2001) 14(4):461–70. 10.1016/S1074-7613(01)00126-1 11336691

[24] Braun D Caramalho I Demengeot J. IFN-alpha/beta enhances BCR-dependent B cell responses. Int Immunol (2002) 14(4):411–9. 10.1093/intimm/14.4.411 11934877

[25] Fink K Lang KS Manjarrez-Orduno N Junt T Senn BM Holdener M Early type I interferon-mediated signals on B cells specifically enhance antiviral humoral responses. Eur J Immunol (2006) 36(8):2094–105. 10.1002/eji.200635993 16810635

[26] Jego G Palucka AK Blanck JP Chalouni C Pascual V Banchereau J. Plasmacytoid dendritic cells induce plasma cell differentiation through type I interferon and interleukin 6. Immunity (2003) 19(2):225–34. 10.1016/S1074-7613(03)00208-5 12932356

[27] Swanson CL Wilson TJ Strauch P Colonna M Pelanda R Torres RM. Type I IFN enhances follicular B cell contribution to the T cell-independent antibody response. J Exp Med (2010) 207(7):1485–500. 10.1084/jem.20092695 20566717 PMC2901065

[28] Bach P Kamphuis E Odermatt B Sutter G Buchholz CJ Kalinke U. Vesicular stomatitis virus glycoprotein displaying retrovirus-like particles induce a type I IFN receptor-dependent switch to neutralizing IgG antibodies. J Immunol (2007) 178(9):5839–47. 10.4049/jimmunol.178.9.5839 17442968

[29] Zhu J Huang X Yang Y. Type I IFN signaling on both B and CD4 T cells is required for protective antibody response to adenovirus. J Immunol (2007) 178(6):3505–10. 10.4049/jimmunol.178.6.3505 17339445

[30] Moir S Malaspina A Pickeral OK Donoghue ET Vasquez J Miller NJ Decreased survival of B cells of HIV-viremic patients mediated by altered expression of receptors of the TNF superfamily. J Exp Med (2004) 200(7):587–99. 10.1084/jem.20032236 PMC2213289 15508184

[31] Battegay M Moskophidis D Waldner H Brundler MA Fung-Leung WP Mak TW Impairment and delay of neutralizing antiviral antibody responses by virus-specific cytotoxic T cells. J Immunol (1993) 151(10):5408–15. 7693811

[32] Pinschewer DD Perez M Jeetendra E Bachi T Horvath E Hengartner H Kinetics of protective antibodies are determined by the viral surface antigen. J Clin Invest (2004) 114(7):988–93. 10.1172/JCI200422374 15467838 PMC518669

[33] Ciurea A Hunziker L Klenerman P Hengartner H Zinkernagel RM. Impairment of CD4(+) T cell responses during chronic virus infection prevents neutralizing antibody responses against virus escape mutants. J Exp Med (2001) 193(3):297–305. 10.1084/jem.193.3.297 11157050 PMC2195917

[34] Fung-Leung WP Kundig TM Zinkernagel RM Mak TW. Immune response against lymphocytic choriomeningitis virus infection in mice without CD8 expression. J Exp Med (1991) 174(6):1425–9. 10.1084/jem.174.6.1425 1683893 PMC2119037

[35] Moskophidis D Cobbold SP Waldmann H Lehmann-Grube F. Mechanism of recovery from acute virus infection: treatment of lymphocytic choriomeningitis virus-infected mice with monoclonal antibodies reveals that Lyt-2+ T lymphocytes mediate clearance of virus and regulate the antiviral antibody response. J Virol (1987) 61(6):1867–74. 3494855 10.1128/jvi.61.6.1867-1874.1987 PMC254192

[36] Steinhoff U Muller U Schertler A Hengartner H Aguet M Zinkernagel RM. Antiviral protection by vesicular stomatitis virus-specific antibodies in alpha/beta interferon receptor-deficient mice. J Virol (1995) 69(4):2153–8. 7884863 10.1128/jvi.69.4.2153-2158.1995 PMC188883

[37] Cumano A Rajewsky K. Structure of primary anti-(4-hydroxy-3-nitrophenyl)acetyl (NP) antibodies in normal and idiotypically suppressed C57BL/6 mice. Eur J Immunol (1985) 15(5):512–20. 10.1002/eji.1830150517 3873342

[38] Hunziker L Recher M Macpherson AJ Ciurea A Freigang S Hengartner H Hypergammaglobulinemia and autoantibody induction mechanisms in viral infections. Nat Immunol (2003) 4(4):343–9. 10.1038/ni911 12627229

[39] Kawamoto H Sakaguchi K Takaki A Ogawa S Tsuji T. Autoimmune responses as assessed by hypergammaglobulinemia and the presence of autoantibodies in patients with chronic hepatitis C. Acta Med Okayama (1993) 47(5):305–10. 8273454 10.18926/AMO/31582

[40] Odermatt B Eppler M Leist TP Hengartner H Zinkernagel RM. Virus-triggered acquired immunodeficiency by cytotoxic T-cell-dependent destruction of antigen-presenting cells and lymph follicle structure. Proc Natl Acad Sci U S A (1991) 88(18):8252–6. 10.1073/pnas.88.18.8252 1910175 PMC52485

[41] Hatzi K Nance JP Kroenke MA Bothwell M Haddad EK Melnick A BCL6 orchestrates Tfh cell differentiation via multiple distinct mechanisms. J Exp Med (2015) 212(4):539–53. 10.1084/jem.20141380 25824819 PMC4387288

[42] Good-Jacobson KL Szumilas CG Chen L Sharpe AH Tomayko MM Shlomchik MJ. PD-1 regulates germinal center B cell survival and the formation and affinity of long-lived plasma cells. Nat Immunol (2010) 11(6):535–42. 10.1038/ni.1877 20453843 PMC2874069

[43] Frebel H Nindl V Schuepbach RA Braunschweiler T Richter K Vogel J Programmed death 1 protects from fatal circulatory failure during systemic virus infection of mice. J Exp Med (2012) 209(13):2485–99. 10.1084/jem.20121015 23230000 PMC3526355

[44] Nie Y Waite J Brewer F Sunshine MJ Littman DR Zou YR. The role of CXCR4 in maintaining peripheral B cell compartments and humoral immunity. J Exp Med (2004) 200(9):1145–56. 10.1084/jem.20041185 15520246 PMC2211858

[45] Chen J Trounstine M Alt FW Young F Kurahara C Loring JF Immunoglobulin gene rearrangement in B cell deficient mice generated by targeted deletion of the JH locus. Int Immunol (1993) 5(6):647–56. 10.1093/intimm/5.6.647 8347558

[46] Zinkernagel RM LaMarre A Ciurea A Hunziker L Ochsenbein AF McCoy KD Neutralizing antiviral antibody responses. Adv Immunol (2001) 79:1–53. 10.1016/S0065-2776(01)79001-3 11680006 PMC7130890

[47] Zinkernagel RM Leist T Hengartner H Althage A. Susceptibility to lymphocytic choriomeningitis virus isolates correlates directly with early and high cytotoxic T cell activity, as well as with footpad swelling reaction, and all three are regulated by H-2D. J Exp Med (1985) 162(6):2125–41. 10.1084/jem.162.6.2125 3877779 PMC2187995

[48] Ciurea A Klenerman P Hunziker L Horvath E Senn BM Ochsenbein AF Viral persistence in vivo through selection of neutralizing antibody-escape variants. Proc Natl Acad Sci U S A (2000) 97(6):2749–54. 10.1073/pnas.040558797 10688894 PMC16001

[49] Silva-Barrios S Smans M Duerr CU Qureshi ST Fritz JH Descoteaux A Innate immune B cell activation by leishmania donovani exacerbates disease and mediates hypergammaglobulinemia. Cell Rep (2016) 15(11):2427–37. 10.1016/j.celrep.2016.05.028 27264176

[50] Allen CD Ansel KM Low C Lesley R Tamamura H Fujii N Germinal center dark and light zone organization is mediated by CXCR4 and CXCR5. Nat Immunol (2004) 5(9):943–52. 10.1038/ni1100 15300245

[51] Bannard O Horton RM Allen CD An J Nagasawa T Cyster JG. Germinal center centroblasts transition to a centrocyte phenotype according to a timed program and depend on the dark zone for effective selection. Immunity (2013) 39(5):912–24. 10.1016/j.immuni.2013.08.038 24184055 PMC3828484

[52] Moorman JP Zhang CL Ni L Ma CJ Zhang Y Wu XY Impaired hepatitis B vaccine responses during chronic hepatitis C infection: involvement of the PD-1 pathway in regulating CD4(+) T cell responses. Vaccine (2011) 29(17):3169–76. 10.1016/j.vaccine.2011.02.052 21376795 PMC3090659

[53] Hannum LG Haberman AM Anderson SM Shlomchik MJ. Germinal center initiation, variable gene region hypermutation, and mutant B cell selection without detectable immune complexes on follicular dendritic cells. J Exp Med (2000) 192(7):931–42. 10.1084/jem.192.7.931 11015435 PMC2193308

[54] Schmittgen TD Livak KJ. Analyzing real-time PCR data by the comparative C(T) method. Nat Protoc (2008) 3(6):1101–8. 10.1038/nprot.2008.73 18546601

[55] Chabot S Fakhfakh A Beland K Lamarre A Oldstone MB Alvarez F Mouse liver-specific CD8(+) T-cells encounter their cognate antigen and acquire capacity to destroy target hepatocytes. J Autoimmun (2013) 42:19–28. 10.1016/j.jaut.2012.10.002 23137675 PMC4465814

[56] Lopez-Macias C Kalinke U Cascalho M Wabl M Hengartner H Zinkernagel RM Secondary rearrangements and hypermutation generate sufficient B cell diversity to mount protective antiviral immunoglobulin responses. J Exp Med (1999) 189(11):1791–8. 10.1084/jem.189.11.1791 10359583 PMC2193076

