# Supplementary figures and images for: Type I Interferon Impairs Specific Antibody Responses Early during Establishment of LCMV Infection
Source: Front Immunol. 2016 Dec 5;7:564. doi: 10.3389/fimmu.2016.00564 (PMC5136549; doi:10.3389/fimmu.2016.00564)

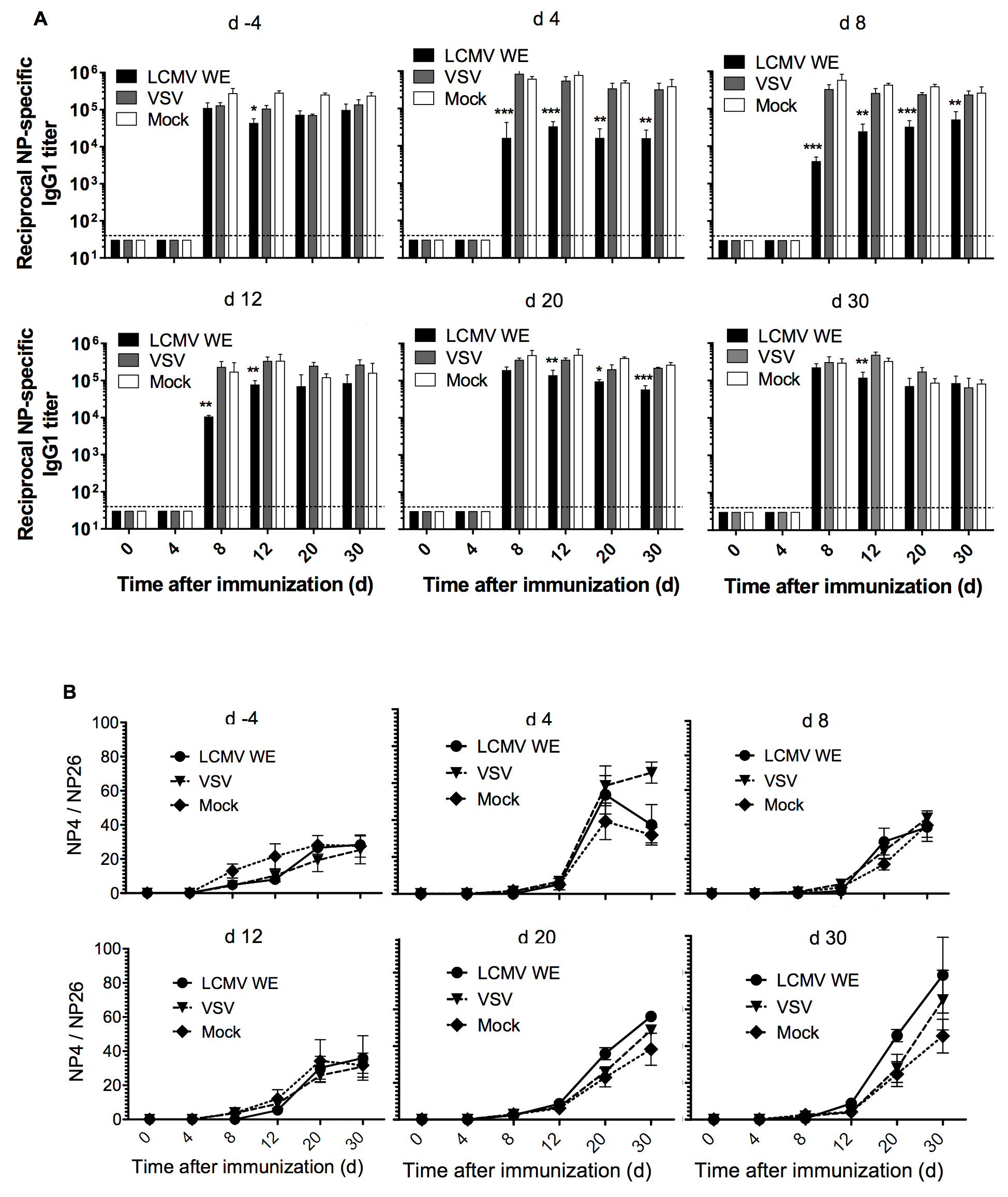

Supplement: Figure S1 — Kinetics of the LCMV WE-dependent impairment of the NP-specific Ab response. B6 mice (four per group) were infected with LCMV WE (black), VSV Ind (gray), or mock infected (white). Mice were then immunized with an i.p. injection of NP53-CGG in alum following a varying immunization schedule (from d-4 to d30). (A) NP-specific IgG1 response in each differentially timed experiment monitored by ELISA. (B) ELISA plates were coated with NP4-BSA or NP26-BSA and high affinity Ab responses were measured as a ratio of Abs binding to NP4-BSA versus the total anti-NP IgG1 response binding to NP26-BSA in each differentially timed experiment. Statistical analysis was performed by individual T-tests between experimental groups and the mock-infected group. *p < 0.05, **p < 0.01, and ***p < 0.001. [file Image_1.TIFF]
